# Editorial: Association Between Human Cancers and Small DNA Tumor Viruses

**DOI:** 10.3389/fonc.2020.592643

**Published:** 2020-09-18

**Authors:** Mauro Tognon

**Affiliations:** Section of Experimental Medicine, Department of Medical Sciences, School of Medicine, University of Ferrara, Ferrara, Italy

**Keywords:** cancer, HPV, PyV, viral oncogenesis, therapy, diagnosis, prognosis

It has been estimated that ~30% of human tumors are linked to oncogenic viruses. Different human malignancies, including oropharyngeal carcinomas, hepatomas and brain tumors, have been found to be associated to small DNA tumor viruses, such as human papillomaviruses (HPV), hepatitis B virus (HBV) and polyomaviruses (PyV), respectively (Rotondo et al.; Zhou et al.). Some small DNA tumor viruses, which are ubiquitous in human populations, may represent a risk for the cancer onset in those patients with immune system impairments. The detection of small DNA tumor viruses in patients affected by cancers and understanding of their mechanism of cell transformation may allow precise treatment options, such as surgery, radiotherapy, immunotherapy, and pharmacotherapy with specific antineoplastic agents and biological drugs. Indeed, in recent years the precision/personalized medicine improved the patient's outcome. Investigations on the association between human cancers and small DNA tumor viruses have become significant for the scientific research in the field of oncology and virology. Data obtained in this oncological area are already employed to address therapeutic strategies to selected targets. In this Research Topic, a collection of reviews was published with the aims to favor (i) the spread of the basic knowledge of this field and (ii) the translational medicine for the distinct cancer types.

In an overview on JC polyomavirus (JCPyV), Del Valle and Piña-Oviedo, summarized the association of JCPyV with the devastating neurological disease of the central nervous system, named progressive multifocal leukoencephalopathy (PML), and with medulloblastomas and gliomas of different histotypes, together with the biology and physiopathology of this PyV. Provenzano and Allayeh in their opinion article report on the association between prostate cancer (PCa) and BK polyomavirus (BKPyV), as well as other PyVs. A general overview is provided about suitable and feasible technologies to be used for PCa diagnosis/prognosis. Specifically, this article addresses the importance of isolating and analyzing the biomarkers in liquid biopsies. Authors indicate that these new technical approaches seem to be promising for cancer diagnosis, prognosis and prediction. Kervarrec et al. focused their review on the Merkel cell carcinoma (MCC) and Merkel cell polyomavirus (MPyV). MCC is a primary neuroendocrine carcinoma of the skin, whereas MCPyV is a new human polyomavirus (HPyV), which DNA was found be integrated into the host genome leading to MCC oncogenesis. The nature of the cell in which MCC oncogenesis occurs is actually unknown. So far, several hypotheses have been postulated. For instance, epithelial as well as fibroblastic or B-cell origin of MCC has been proposed mainly based on phenotype similarities shared by MCC and these potential ancestries. The Touzé's group provides a comprehensive review of the current knowledge of the MCC histogenesis. Rotondo et al. in their review on the association between human cancers and the oncogenic polyomavirus Simian virus 40 (SV40) report on new data obtained with indirect ELISA tests with synthetic peptides as mimotopes/specific SV40 antigens. Serum samples from oncological patients had a higher prevalence of antibodies against SV40 compared to healthy subjects. These innovative immunological results strengthened the association between SV40 and specific human tumors, while at the same time allowed to circumvent controversies arisen on data obtained by early molecular biology studies. In his review, Gheit reports on the association between mucosal/cutaneous cancers and oncogenic human papillomaviruses (HPVs). More than 200 different HPVs have been listed so far. Based on epidemiological data, some HPVs are classified as high-risk (HR) HPV types. HPVs are the etiological agents of anogenital cancers, while a HPV subset of head and neck cancers. The cutaneous HPV types are present on the surface of the skin in the general population. However, beta HPVs seem to play a role, together with ultraviolet (UV) radiation in non-melanoma skin cancer (NMSC). Molecular mechanisms showed that HPV oncoproteins E6/E7 alter pathways involved in the host immune response to establish a persistent infection and by promoting cellular transformation. Zhou et al. reported on the HPV strategy to evade the immunological system. They recalled that HPV persistent infection initiates ~5% of all human cancers, including cervical and oropharyngeal cancers. HPV vaccines prevent HPV infection, but do not eliminate existing HPV infections. HPV-induced hyperproliferation renders epithelial cells less sensitive to immune attack, and impacts upon the efficiency of the local immune system. These observations have significance for the design of therapeutic HPV cancer immunotherapies.

In conclusion, authors of this Research Topic addressed their reports on the mechanisms of cell transformation operated by small DNA tumor viruses, such as PyVs and HPVs. These reviews indicated novel diagnostic tools, new therapeutic targets and strategies to be transferred to the clinical practice with the aim to cure tumors associated with oncogenic viruses.

## Author Contributions

The author confirms being the sole contributor of this work and has approved it for publication.

## Conflict of Interest

The author declares that the research was conducted in the absence of any commercial or financial relationships that could be construed as a potential conflict of interest. The handling editor declared a shared affiliation with the author at time of review.

